# A Procedural Justice Theory Approach to Police Engagement with Victim-Survivors of Rape and Sexual Assault: Initial Findings of the ‘Project Bluestone’ Pilot Study

**DOI:** 10.1007/s43576-022-00056-z

**Published:** 2022-05-04

**Authors:** Katrin Hohl, Kelly Johnson, Sarah Molisso

**Affiliations:** 1 City, University of London, London, UK; 2 Durham University, Durham, UK

**Keywords:** Procedural justice theory, Sexual violence, Victims, Policing

## Abstract

In England and Wales, public trust in the police has been damaged by a series of police failings in rape and sexual assault investigations, officer sexual offending, and a police culture of misogyny. Feminist scholars have analysed why police investigations of rape and sexual assault cases rarely result in a charge and documented the poor experiences many victim-survivors have of the police process. In this article, we outline how this scholarship may be integrated into procedural justice theory to advance our understanding of the impact of how officers engage with victim-survivors on their feelings of the status and value as survivors of sexual violence within the nation and society police represent, as well as on their trust in the police and willingness to (continue) engaging with police, or report future victimisation. We present tentative evidence from a pilot study (‘Project Bluestone’) in one English police force that suggests a feminist scholarship informed Procedural Justice framework is a promising tool for assessing and improving police practice in engaging with victim-survivors of rape and sexual assault. The article concludes with directions for future research.

## Introduction

How police respond to sexual violence, in particular sexual violence against women and girls, has become a litmus test for public trust in the police. In the UK, the rape and murder of Sarah Everard by a serving police officer, and the heavy-handed policing of peaceful vigils in her memory, attended mostly by women, have measurably eroded public trust in the police. Two years prior, 70% of the UK public felt “the police were doing a good job” (a widely used measure of public trust), whilst only 54% said so in February 2022, the lowest on record (YouGov, 2022). The data (ibid.) further show that key shifts in this indicator occurred following media coverage of the Sarah Everard case and the Metropolitan Police’s subsequent ill-conceived attempts – and failure – to reassure women of their safety, and a string of further revelations of officer sexual offending and misogyny pervading police culture. Victim-survivors, and those campaigning on their behalf, have long denounced endemic misogyny in policing and the police not taking sexual violence seriously enough, as evidenced by low charge rates (only 1.6% of recorded rapes in England and Wales result in a suspect being charged; George & Ferguson, 2021) and the police process being experienced as a ‘secondary victimisation’ for many victim-survivors, further exacerbating the trauma of the rape or sexual assault (‘rape’ hereafter) (Campbell & Raja, 1999; Stanko, 1985). Experiences of secondary victimisation can result from police officers behaving in ways that signal to the victim-survivor disbelief, disinterest, victim-blaming, or a lack of empathy, care, or respect towards victim-survivors (Campbell, 2005). Unnecessarily intrusive investigation of the victim-survivors’ backgrounds, including their school and medical records, and ‘digital strip searches’ of mobile phones and social media only compound this experience (Dodge et al., 2019).

In this article, we argue procedural justice theory provides a useful theoretical framework for explaining why poor police treatment, specifically treatment that lacks the components of procedural fairness, does not merely result in dissatisfaction with police, but can cause significant harm to victim-survivors. Procedural justice theory further provides explanations for why police behaviour that does not accord with the principles of procedural fairness undermines public trust in the police, and by extension police legitimacy – which, in turn, reduces people’s willingness to engage with police, for example, by supporting the police investigation, or reporting rape in the first place (Tyler, 2006; Tyler & Huo, 2002; see also Bottoms & Tankebe, 2012). Procedural justice theory has been tested extensively in a variety of national contexts (Donner et al., 2015; Jackson, 2018). However, thus far the focus has overwhelmingly been on scenarios of police-initiated contact where a person’s options for non-cooperation are limited, for example, in the context of stop and search. When applied to situations of public-initiated contact, existing scenarios are typically built around willingness to report low-level crimes and cooperation when little is at stake (e.g. pickpocketing), or only ask about willingness to report ‘a crime’ in the abstract.

Rape is an iconic offence in the measure of women’s equality in society (Hohl & Stanko, 2015), and how police treat complaints and complainants of rape might thus be considered of particular interest in testing the universality of procedural justice theory. Yet, surprisingly little research has explored the applicability of the conceptual framework in the context of victims’ perceptions of police fairness, even less specifically with victims of rape (Elliott et al., 2014; Lorenz, 2017; Lorenz & Jacobsen, 2021; Murphy & Barkworth, 2014). This article contributes to this emerging body of literature by exploring how a procedural justice theory framework might be deployed to assess and improve police practice in their engagement with rape victim-survivors, with the aim of improving police interactions and rebuilding victim-survivor trust in the police. In doing so, the article departs from prior studies in that it does not test the empirical validity of procedural justice theory within a general population sample. Instead, its contribution is two-fold. Theoretically, it begins to integrate the knowledge of victim-survivors’ experiences of police interactions developed through decades of feminist research into the procedural justice theory model to advance understanding of the police relationship with and impact on victim-survivors. Empirically it explores, in the limited ways a small pilot study allows, the practical use of such an understanding for assessing and improving police practice in engaging with victim-survivors over the course of a police rape investigation.

## Theoretical Framework

Underlying procedural justice theory is Tyler and Lind’s *group-value model*. The model suggests that how authority figures in our group (e.g. society) treat us has implications for our feelings of value and status within this group, with implications for our self-worth and self-identity (Sunshine & Tyler, 2003; Tyler & Lind, 1992). Specifically, we draw such inferences about our group status and self-identity from whether police, as representatives of the state, society, and law, interact with and act towards us with *procedural fairness*. Procedural fairness is often defined as being constituent of four key components: voice; dignity and respect; neutrality; and trustworthy motives (Bradford et al., 2021; Tyler, 2006). *Voice*, sometimes called ‘active participation’ means that we are given the opportunity to tell ‘(our side of) the story’, that police listen to our (side of) the story and take this into account when making decisions relevant to us. Treating people with *dignity and respect* is a second component. *Neutrality* encompasses unbiased decision-making, and includes treating everyone equally, regardless of their race, gender, religion, or class. *Trustworthiness* forms the fourth component and refers to perceptions of whether officers have trustworthy motives and show concern for the wellbeing of those impacted by their decisions (Rosenbaum et al., 2017; Tyler, 2006). How these components are defined and relate to each other, public trust, and police legitimacy have largely been explored through operationalising them in general population surveys and testing their overlaps with and relationships to other concepts through structural equation modelling (Jackson, 2018).

A small number of procedural justice studies focus on rape victim-survivors specifically. These empirically corroborate the link between victim-survivor experience of police behaviour and their feelings of self-worth and identity, and the impact of police interactions on their recovery from sexual violence. These studies evidence the importance and possibility of what Wemmers (2008) terms ‘therapeutic jurisprudence’, achieved by victim-survivors having voice or control within the criminal justice process, receiving support, and feeling recognised, empowered, and respected by justice authorities. Officer behaviour that acknowledges that wrong and harm have been done to the victim-survivor, demonstrates non-blaming attitudes and compassionate listening, and treats victim-survivors as whole and valued persons demonstrably contributes to the validation of victimisation experiences, reducing the harm and trauma associated with the crime and the police process, thereby aiding victim-survivors therapeutically. Conversely, officer behaviour that denies voice, does not acknowledge harm, is judgemental, uncaring, intimidating, or insensitive, is found to have negative or ‘anti-therapeutic’ effects, is engendering secondary victimisation (Elliott et al., 2011, 2014; Lorenz, 2017; Wemmers, 2008, 2013).

The logic of procedural justice theory continues as follows. Perceptions of procedural fairness shape trust in the police. Trust in the police in the words of Bradford (2020, p.177–178) means “an individual trusts a person or institution when they willingly place valued outcomes – for example their security or freedom – in the hands of that actor or institution on the basis of a belief that they have the right intentions and are competent to perform relevant actions”. Perceptions of police procedural fairness have been found to be a stronger predictor of public trust in the police than a favourable outcome of the police interaction (Tyler, 2006). This is consistent with the feminist concept of ‘kaleidoscope justice’ which presents a complex, multifaceted, and fluid understanding of what justice means within the lived experience of rape victim-survivors. This includes conceptualising justice as involving ‘consequences’ (seeing the perpetrator held accountable and punished) and ‘prevention’, aligning with common notions of criminal justice-based ‘outcome justice’. However, kaleidoscope justice also includes themes of ‘recognition’, ‘dignity’, ‘voice’, and ‘connectedness’ as justice, which encompass victim-survivors receiving societal validation that what has happened is ‘true’ and acknowledges the wrong and harm they have experienced (McGlynn & Westmarland, 2019), echoing the procedural fairness components identified by Wemmers (2008, 2013), Elliot et al. (2014), and Lorenz (2017).

Returning to the logic of procedural justice theory, trust in the police has been shown to inform perceived police legitimacy, our willingness to cooperate with police, and comply with the law (Jackson et al., 2013). At present, few rape victim-survivors act in ways that would suggest high levels of trust in the police when it comes to reporting rape – whereby they willingly place valued outcomes such as their search for justice and securing their and others’ safety into the hands of police on the basis of believing that police have ‘good’ intentions and will competently respond and investigate (Bradford, 2020). It is estimated that only a fraction of rapes come to police attention, and in 65% of cases victim-survivors stop ‘cooperating’ (i.e. engaging) with police and withdraw from the investigation (Wunsch et al., 2021). Furthermore, willingness to report future victimisation has been a main focus of existing applications of procedural justice theory to victims of crime, with studies consistently finding evidence of procedurally fair treatment being predictive of future willingness to report (Lorenz & Jacobsen, 2021; Murphy & Barkworth, 2014).

In sum, procedural justice theory provides an empirically tested model for understanding the impact of police encounters on our sense of self-worth, value, and status within society, as well as on our trust in the police, perceived police legitimacy, and consequently, our willingness to cooperate with police. A relatively small body of victim-survivor studies provides some evidence of the principles of procedural justice similarly applying in the context of rape. However, feminist research has provided a far more diverse and nuanced understanding of the experience of reporting rape to the police, and its impact on victim-survivors, and may be useful for expanding and consolidating procedural justice theory. The pilot study presented in this article explores the practical application of such an understanding for assessing and improving police practice in engaging with victim-survivors over the course of a police rape investigation.

## The Pilot Study

The ‘Project Bluestone’ pilot study was conducted in 2021 with Avon and Somerset Police, a medium-sized police force in England with a mix of urban and rural areas (Hohl and Stanko*, this issue*).^1^ The findings reported in this article draw on data collected in six focus groups with police officers and two focus groups with Independent Sexual Violence Advocates (ISVAs) working in the local police force area. Police focus groups participants included a variety of officer ranks and roles, from frontline response constables to detective inspectors. The ISVA focus groups were designed to capture ISVA perceptions of how victim-survivors accessing their service experienced police contact throughout the investigation of their case. All fieldwork was conducted during a Covid-19 lockdown which meant focus groups took place via an online video-conferencing app (see Richardson et al., 2021, for a review of this mode of data collection). Participants were recruited by the police force and the ISVA service respectively, using a non-probability sampling approach. Consequently, findings are not generalisable to all officers or all ISVAs. Each focus groups consisted of five to eight participants. One author led the focus groups while, with the consent of participants, two of the authors took detailed notes (video and audio recording was not permitted). Notes were then coded, and key themes identified and then assessed against the four elements of procedural fairness – voice, dignity and respect, neutrality, and trustworthiness. Emerging findings were presented back to police and ISVA participants as well as to a lived experience panel of rape survivors in a series of consultative meetings, and their feedback was used to inform the findings, recommendations, and practice products developed for this study.

## Findings

### Voice

Victim-survivors not feeling heard in their engagements with the police emerged as a key theme amongst ISVA participants. When making a report of rape, victim-survivors would be asked to repeat their account on multiple occasions to a series of different officers with different functions within the early stages of the investigation, often in unnecessary detail. Such multiple handovers of the case and having to repeat the same information several times necessitated by police organisational structures and processes likely contribute to a sense of not having been listened to. Further, ISVA participants reported victim-survivors felt they had little control over or understanding of what was happening to them and how the process ‘worked’, negatively impacting victim-survivors’ sense of active participation and citizenship in the investigation. Examples included victim-survivors feeling they were rushed by officers into particular actions for the benefit of the investigation, such as giving their video-recorded interview or agreeing to third-party disclosure requests (e.g. medical, social services, school records, or counselling notes), at the expense of their wellbeing, and/or when they did not fully understand the processes or implications of these actions. Additionally, ISVA participants reflected officers would often use inaccessible language and police jargon in their communications, resulting in victim-survivors not fully understanding what has been said or what actions had been taken, especially in circumstances where English is not their first language. Moreover, victim–survivors reported feeling officers sometimes did not take the time to understand their wishes and views, or provide them with space to ask questions, in effect silencing the victim-survivor’s voice in the process. Examples included officers interrupting or cutting victim––survivors off when providing their account, or concentrating on criminal justice outcomes without exploring what alternative outcomes might be available to or desired by the victim-survivor, such as civil orders, providing intelligence, or other forms of safeguarding and support.

### Dignity and Respect

Officer focus groups revealed significant variability between officers in interpersonal skills and confidence when engaging with rape victim-survivors. Both officer and ISVA focus groups provided examples of good practice, such as officers being mindful of body language (e.g. not towering over a seated victim-survivor) as well as examples of poor practice, including officers misgendering a victim-survivor despite them clearly stating their chosen pronouns. A key driver for this variability in the quality of police engagement, in the view of participating officers, were significant workload pressures. This, officers reported, was compounded by severely limited resources and lack of clinical support and supervision, causing high levels of occupational stress, burnout, and ‘empathy fatigue’. These challenging circumstances, police participants reflected, meant often officers, despite having all the right intentions, had limited capacity to sufficiently engage with victim-survivors and meet their communication and support needs, all of which are central to instilling a sense of dignity and respect in victim-survivors. Further, officers felt they had limited training and capacity for tailoring their engagement to different victim contexts and needs. For example, whilst there were officers with specialist expertise in engaging with sex workers praised both by officers and ISVAs, ISVAs noted such specialism was absent for other victim-survivors, such as those from ethnically or culturally minoritised groups, or those with learning disabilities. This finding points to the need for protocols that ensure accessibility and sensitivity, and thus dignity and respect, for *all* victim-survivors of sexual violence. ISVA participants further stressed the significance of officers’ actions, tone of voice, or choice of words being amplified to victim-survivors because of the police’s position of authority and power, especially relative to rape complainants. ISVA participants felt officers did not always appreciate the effect their position of authority can have on victim-survivors and failed, for example, to understand that victim-survivors can be impacted and undermined by officers’ off-hand comments, or a disengaged tone of voice. This suggests that whilst police symbolic power is generally accepted within the academic literature (Bradford, 2014), and apparent to ISVAs, officers may not always be aware of, acknowledge, or act in ways recognisant of this.

### Neutrality

Our analysis of the sequencing of the police investigation found that the ordering of investigative steps undermined its procedural neutrality. For cases reported 30 days or more after the alleged offence (deemed ‘non-recent’ by the force), the so-called ‘Bluestone Unit’ would support the victim-survivor, but also take the victim-survivors’ initial account, formal (video-recorded) statement, and request their consent for police to access third-party and digital disclosure materials, to be reviewed by the unit. Cases would only be transferred to the Criminal Investigation Department (CID) for a full investigation, including potential arrests and interviewing of suspects, if and when the investigation of the victim-survivor’s account and all victim-survivor-related background material had been completed. The Bluestone Unit had originally been established to provide dedicated, specialised, and quicker service for victim-survivors of rape, and as such was well-intended. Yet, the Bluestone Unit was, in effect, a ‘victim-survivor credibility investigation unit’, frontloading and over-focusing the investigation towards victim-survivor credibility. This well-intended, yet ill-conceived set-up is indicative of insufficient consideration given to procedural fairness – in particular, whether starting an investigation by examining the victim-survivor’s credibility might undermine neutral decision-making. This approach could well suggest to victim-survivors police suspiciousness of their truthfulness and is reminiscent of Jordan’s (2004) research addressing the lack of credibility afforded to ‘the word of a woman’ and the pernicious rape myth that false rape allegations are frequent.

Within the officer focus groups there was also some debate over what ‘neutrality’ meant within rape investigations. Some officers felt this meant they should avoid communicating to victim-survivors whether they believed their account, and position themselves as impartial evidence gatherers, not ‘arbiters of truth’. Other officers challenged this view, suggesting that such ‘neutral’ behaviour could be read by victim-survivors as detachment and disbelief, and felt it important to communicate to victim-survivors that they were believed and, consequently, their report taken seriously. Doing so, they contended, would not undermine neutrality of their case decision-making and functioned as a supportive enabler for victim-survivors engaging with the criminal justice process.

### Trustworthy Motives

‘Trustworthy’ motives involve officers having the wellbeing or best interests of victim-survivors at heart. A recurring theme in ISVA participant reflections was the inconsistent support and communication offered by officers, leaving many victim-survivors feeling they had not been afforded appropriate consideration. Examples of poor communication included officers failing to call on agreed dates and failing to provide regular, meaningful updates, or communicating important pieces of information inappropriately. For instance, police decisions to take ‘no further action’ (i.e. drop the case) were at times communicated to victim-survivors via text message. At times officers would phone victim-survivors late at night with abrupt updates because this suited their shift pattern without considering the impact of an unexpected late night police call on victims-survivors, or that ISVA services were unavailable during those times to help victim-survivors process the update. Both ISVA and officer participants felt such ill-considered communications may be a result of workload pressures, limited resources, or administrative delays, rather than ill will. Yet, officers agreed with the ISVA focus groups’ finding that victim-survivors would understandably interpret such actions as being indicative of police not having the victim-survivors wellbeing at heart, or signalling a lack of interest in their case.

## Implementation of Findings

As a result of the findings and recommendations developed through this research, Avon and Somerset Police have piloted a number of practical changes to improve their engagement with victim-survivors. As a central component of the implementation, researchers met regularly with officers to discuss the findings, the logic of procedural justice theory informed by feminist literature on victim-survivor experience, the symbolic power of police actions, and to iteratively and collaboratively develop practical products and processes to put these ideas into practice, as described below.

### Voice

All victim-survivors now receive a sexual offences information booklet, developed by the authors in consultation with the officers, ISVAs, and survivors to empower victim-survivors by providing information about the criminal justice process and victim-survivors’ rights. The booklet is provided at the initial contact and is available on the police force’s website for those considering making a report. Newly created ‘Engagement Officer’ roles have reduced the number of handovers that victim-survivors experience, aimed at improving rapport and decreasing the number of times victim-survivors are asked to repeat their account. Additionally, this new role aims to increase officer capacity to meet victim-survivors before the video-recorded interview, to discuss the interview process, provide the opportunity to ask questions or voice concerns, and engender a greater sense of agency in victim-survivors.

### Trustworthiness, Dignity, and Respect

A ‘victim communication plan’ template (again developed by the authors following participant consultation) is now used by Engagement Officers at the start of the investigation to agree and record victim-survivors’ preferences on mode of contact (e.g. call, text message, or via their ISVA), progress updates, and any relevant safeguarding, additional needs, or privacy concerns. Officers are now piloting use of a ‘No Further Action’ letter template, developed by the authors, to aid officers in writing these letters sensitively. The template now explicitly thanks victim-survivors for trusting the police with their report, requires a detailed explanation of the reasons why ‘no further action’ has been taken, clearly explains the ‘Victim’s Right to Review’ scheme and complaints process, and signposts to relevant support services. As a result of these changes, the ISVA service has already reported a significantly improved police-ISVA working relationship, which they believe is improving support for victim-survivors. Further, the police service is now using its social media, participating in newspaper and television interviews, and taking part in YouTube videos with ISVAs to openly own past shortcomings in victim-survivor engagement, explain their new approach, and demonstrate genuine passion for supporting victim-survivors, and in doing so communicate trustworthy motives.

### Neutrality

The ‘Bluestone Unit’ has been disbanded and replaced with a ‘geographic improvement’ pilot. Resultantly, the procedural frontloading of investigating the victim-survivor’s credibility through third party and digital material has reduced. Instead, the new model emphasises focussing on arresting, interviewing, and investigating the suspect at the earliest opportunity, and offender risk management and disruption methods. New, joint training between the police and CPS is designed to empower officers to employ a ‘reflexive’ approach to requesting third-party and digital material relating to the victim-survivor, emphasising that such requests must be clearly proportionate and necessary (i.e. not a ‘fishing expedition'). These measures aim to re-balance the investigation and achieve greater neutrality in police decision making, by limiting any over-reach or over-focus of the investigation on the victim-survivor.

## Discussion

Our findings on police victim-survivor engagement practices are consistent with wider existing literature detailing inconsistent and at times negative victim-survivor criminal justice experiences. These findings clearly map on to the four, overlapping components of procedural fairness: limited and sometimes poor communication from officers during the investigation leaving victim-survivors feeling uninformed and silenced (*voice*); variability in officer interpersonal skills and confidence in victim-survivor engagement resulting in poor rapport between officers and victim-survivors, and variability in officers’ addressing victim-survivors’ intersectional contexts, support needs, and vulnerabilities (*dignity and respect*); a frontloading and over-focus of the investigation on the victim-survivor’s credibility (*neutrality*) (see Horvath & Brown, 2022); and victim-survivors being left questioning whether officers believe their reports, see them as valued citizens (see e.g. McGlynn & Westmarland, 2019) and take their cases seriously (*trustworthy motives*).

Although small in scale, the pilot study permitted us to directly compare officer and ISVA perceptions on the quality of police victim-survivor engagement within the same police force area at the same point in time, generating compelling findings requiring further exploration. Firstly, officers and ISVAs recognised great variability in officer engagement practices, and consequently, in victim-survivor experiences. Both groups agreed officer practices were often shaped by the context of the case, police capacity at the time, and/or the officers involved – suggesting that a victim-survivor experiencing ‘good’ police engagement was more a matter of ‘luck’ than a result of established organisational processes, structures, and culture that enable consistently good victim-survivor engagement. Officers and ISVAs also converged on perceiving the majority of officers as having good intentions towards victim-survivors, however, ISVAs felt officer behaviour in practice could, sometimes unthinkingly, communicate the opposite. Accordingly, ISVA participants emphasised the importance of officers being mindful of their body language and tone of voice, using appropriate language, and taking time to explain the investigative process, to build trust and convey their *trustworthy motives*. Similarly, whilst our ISVA participants felt most officers treated, or sought to treat, victim-survivors with *dignity and respect*, they felt measures to address victim-survivors’ support needs, for example by engaging with the ISVA service, were not implemented well in police practice, despite such victim-survivor support having consistently been shown to improve victim-survivor experience and case outcomes (see Hester & Lilley, 2018).

One significant area of divergence between participants related to police symbolic power and its interrelationship to victim-survivor experience and trust in the police. While ISVA participants were acutely aware of the impact of police interpersonal behaviour on victim-survivors, echoing the group value model underlying procedural justice theory (Sunshine & Tyler, 2003), this theme was notably absent from officer focus groups, and police participants were relatively less able to acknowledge or engage with their positionality and the power inequalities underpinning their interactions with victim-survivors. Nevertheless, when we presented our study findings to police participants using procedural justice theory to frame and evidence the symbolic power of police action, officer feedback suggested this was a ‘light bulb’ moment for many, which appeared to increase officer buy-in towards our recommendations. This observation echoes other research which notes the significant currency procedural justice carries within contemporary policing practice circles (Bradford, 2020), and makes apparent the potential of procedural justice theory for increasing officer recognition and understanding of victim-survivors’ experiences in the context of rape. It cannot be overstated how important it is for officers to understand the aims and purpose of recommended changes in order to avoid implementation failure (see MacQueen & Bradford, 2017).

Another observed benefit to the procedural justice group-value model (Tyler & Lind, 1992) is that it clearly communicates to officers that rape victim-survivors are behaving in entirely expected, rational, and proportionate ways in seeking recognition or validation of their experience in their interactions with officers, and in being affected by the quality of those interactions in their sense of self-worth, trauma, and recovery. Moreover, because the group-value model applies *across* criminal justice contexts, this framing avoids exceptionalising rape victim-survivors, thereby challenging any problematic officer beliefs about rape victim-survivors being uniquely ‘over-sensitive’ or ‘hard work’ if they are deeply impacted by police interactions. We also propose that applying the wider lens of procedural justice to rape investigations may facilitate officers to engage further with aspects such as intersectionality, discrimination, and accessibility in their victim-survivor-engagement practice. A main site of testing of procedural justice theory has been in the context of the stop and search of young black men, where studies have consistently found that disproportionate and discriminatory police practices marked by a lack of procedural fairness signal exclusion to black citizens, and result in reduced police legitimacy and willingness to cooperate and comply with the police within black communities (Jackson et al., 2013). It is important, therefore, to explore further how a procedural justice approach to victim-survivor engagement might differ in the context of different victim-survivor needs, identities, experiences, and positionalities, informed by an examination of diverse victim-survivor perceptions of procedural fairness (e.g. engaging with individuals from racially, culturally, or sexuality-minoritised communities and/or those living with learning difficulties, disabilities, or mental ill-health).

Finally, in the officer focus groups we were struck by the apparent complexity of interpreting and operationalising *neutrality* in police engagement with victim-survivors of rape – emphasising another important avenue for further inquiry. Particularly, officers disagreed about what officer neutrality might and should mean in practice, inclusive of whether it is appropriate for officers to communicate belief to victim-survivors. It is crucial that any such research situates the concept of ‘neutrality’ within existing victim-survivor literature. This literature has consistently found that a victim-survivor’s (continued) participation in the criminal justice process is directly impacted by their perceptions of officer belief (Jordan, 2004; Wemmers, 2013; Wunsch et al., 2021); that rape myths and stereotypes work to discredit victim-survivors and protect suspects with measurable impacts on case attrition (Hohl & Stanko, 2015) and very low conviction rates (George & Ferguson, 2021), making it unclear whether ‘neutrality’ within individual officer decisions or interactions serve to perpetuate unfairness, rather than demonstrating ‘objectivity’ and fairness within this context.

## Conclusion

Procedural fairness has been empirically explored in a variety of contexts, notably in stop and search, traffic stops, and police community engagement (Jackson, 2018). However, to date, only a small number of scholars have explored its significance for victim-survivors of sexual violence (for example, Elliot et al., 2014; Lorenz, 2017; Wemmers, 2008, 2013). Feminist scholarship offers a nuanced understanding of victim-survivors’ experiences and expectations of police interactions and perceptions of justice (for example, Brooks-Hay, 2020; Horvath & Brown, 2022; McGlynn & Westmarland, 2019), yet this literature remains under-utilised within existent work that applies procedural justice to police responses to rape. This article proposes a way forward for the integration of these two bodies of literature. Additionally, our findings from the pilot study provide tentative, face-value evidence that the four components of procedural fairness – voice, dignity and respect, neutrality, and trustworthy motives – can be used to structure assessments of police engagement with victim-survivors and is a promising vehicle for effecting change in police practice, though the effectiveness of these changes is yet to be formally evaluated. As such, this article identifies several pertinent directions for further research. Any such work must be informed by an understanding of the meaning of procedural fairness for victim-survivors of rape in particular, and engage with research on the ongoing relevance of pernicious rape myths in policing, the widespread under-reporting of rape and victim-survivors disengaging from the investigation process, as well as the longstanding over-policing and under-protection of people from minoritised groups (Bowling et al., 2019). These contexts will prove crucial for advancing the conceptual development of procedural fairness and, in turn, the design of quantitative measures (i.e. survey items) and formal testing of procedural justice theory in the context of rape. Most importantly, at a time where current policing responses to rape are demonstrably damaging public trust in the police, a new, innovative approach to reforming policing practice is urgently needed – and expanding procedural justice theory to include feminist scholarship and victim-survivor perspectives appears to be a promising ground through which such change might be achieved.

## Footnotes

1 Operation Soteria Bluestone is a UK Home Office-funded programme designed to improve the investigation of rape and serious sexual offences (RASSO) in England and Wales. It is a unique project which is underpinned by rigorous social science. With multi-disciplined academics located in multiple universities, mixed qualitative and quantitative methods are applied to a five pillared approach to organisational change with police forces, uplifting the capability of more specialist police decision-making in RASSO cases. The research informs policing practice as well as government policy and is set to inform a national change. These research informed pillars pinpoint specific areas for improvement which will form part of the new framework for investigating RASSO: (1) suspect-focused investigations; (2) disrupting repeat offenders; (3) victim engagement as procedural justice; (4) promoting better learning, development, and wellbeing for police officers; and (5) using data more effectively in RASSO investigations. The pathfinder project started in 2021, based in Avon and Somerset Constabulary. Designed by Katrin Hohl and Betsy Stanko, the pillar leads include Kari Davies, Miranda Horvath, Kelly Johnson, Jo Lovett, Olivia Smith, and Emma Williams.
